# Lead, Cadmium, Zinc, and Copper Bioavailability in the Soil-Plant-Animal System in a Polluted Area

**DOI:** 10.1100/tsw.2010.33

**Published:** 2010-02-17

**Authors:** Violina R. Angelova, Radka V. Ivanova, Jivko M. Todorov, Krasimir I. Ivanov

**Affiliations:** ^1^ Department of Chemistry, Agricultural University, Plovdiv, Bulgaria; ^2^ Department of Plant Science, Agricultural University, Plovdiv, Bulgaria

**Keywords:** heavy metals, polluted soils, intake, bioaccumulation, rapeseed, rabbits

## Abstract

A comparative research study on the bioavailability of Pb, Cd, Zn, and Cu in the soil-plant-animal-system was carried out. The connection between the total quantity and the mobile forms of Pb, Cd, Zn, and Cu in soils with different levels of contamination; the transition of these metals into rapeseed; and their assimilation by rabbits fed with a food that consisted mainly of rapeseed was studied. It was established that the absorption of heavy metals by the rapeseed definitely has a selective character, as the affinity towards Zn is most strongly expressed. The accumulation of Pb, Cd, Zn, and Cu in the organs of the rapeseed occurs in the following order: inflorescences > leaves > stems. A direct connection between the quantity of the mobile forms and their accumulation in the plants was not found. The environmental contamination has a significant effect on heavy metal levels and distribution, as the largest quantity of all four elements is accumulated in the kidneys and liver. A well-expressed impact of the level of Cd contamination on the absorption of essential trace metals (Zn and Cu) and their accumulation into some of the organs of the animals was found.

## INTRODUCTION

Toxic metals are natural components of the environment, but human activities, notably industrial and mining processes, have been responsible for the wider diffusion of these elements. The transport, residence time, and fate of pollutants in an ecosystem are serious social concerns. Due to the highly complex behavior of trace elements within an ecosystem, studies are usually conducted separately for air, water, soil, and biota[1,2]. Plants are good soil quality indicators as they directly reflect air quality. Since plants can naturally absorb pollutants from their local environment, their chemical composition can indicate the degree of disturbance when assessed against background values obtained from unpolluted vegetation. Plants adapt to the great variability of chemical properties within their environment, and are an intermediate medium through which trace elements in soil, water, or air move to animals and humans[2].

Cadmium (Cd) and lead (Pb) are nonessential elements that are toxic to plants and animals, while zinc (Zn) and copper (Cu) play essential metabolic roles in plants and animals[3]. It is known that only a small amount of the total Pb in soils may be taken up by plants, and the translocation of Pb from roots to tops is greatly limited[1]. Cd, Zn, and Cu can be more readily taken up by plants and relatively high concentrations may occur in crops for human consumption. The amount of Pb, Cd, and Zn taken up by plants depends on the total amounts in the soil and their availability[4]. The availability of heavy metals is influenced by a wide range of factors, including soil pH, organic matter, carbonates, clay minerals, and oxides[3].

Toxic metals are accumulated in plants, and animals fed with these plants will tend to accumulate toxic metals themselves. Although contamination of animal feed by toxic metals cannot be entirely avoided given the prevalence of these pollutants in the environment, there is a clear need for such contamination to be minimized, with the aim of reducing both direct effects on animal health and indirect effects on human health[5]. Toxic effects of metals have been described in animals under relatively low levels of metal exposure[6] and one of the earliest effects is the disruption of trace element metabolism[7]. Pb interacts with calcium (Ca) in the nervous system to impair cognitive development. Cd interacts with Ca in the skeletal system to produce osteodystrophies. Pb replaces Zn on heme enzymes and Cd replaces Zn on metallothionein.

The need to reduce toxic metal contamination in animal feed in fact poses a significant problem for agricultural regions located in more-or-less industrialized areas in which animals are reared on locally produced feed. There are many such regions worldwide, including in Bulgaria. The district near the Rhodopi Mountain and the Plovdiv Field has been exposed to the influence of the Non-ferrous Metal Works (NFMW) for a long period of time. In the vicinity of the NFMW near Plovdiv, more than 2,100 ha have been polluted by heavy metals. It is estimated that approximately 460 tons of dust, containing mainly Pb, with less Zn and Cd, have been annually released into the atmosphere for more than 40 years[8,9]. As a result of the new cleansing facilities built in the NFMW, however, the aerosol pollution in the district has been reduced to a minimum. This makes it extremely appropriate to examine the connection between soil and plants regarding the assimilation of heavy metals. This area possesses high levels of heavy metals in soils and in vegetation[10,11,12].

One type of crop grown in such districts is rapeseed[13,14,15,16)]. Its cultivation is mainly for the production of oil, which is widely applied in the food, soap, textile, paint, and varnish industries, as well as in metallurgy and machine building for production of valuable lubrication oils. Winter rapeseed has high nutritional value and thus can be used for green fodder. Valuable protein groats are obtained through the processing of its seeds, which may then be used in the production of combined fodder.

The transition of heavy metals from this type of fodder into the nourished animals is studied in detail in pigs, cattle, sheep, and goats[17,18,19,20,21,22,23,24,25,26]. Very little is known, however, about the heavy metal content and localization in the organs of rabbits.

The aim of the present work was to evaluate the bioavailability of heavy metals in the soil-plant-animal system. This involved an investigation of the connection between the total quantity and the mobile forms of Pb, Cd, Zn, and Cu in soils of varying levels of contamination; the transition of these metals into rapeseed; and their ingestion by rabbits fed with a rapeseed-dominant food mixture.

## MATERIALS AND METHODS

### Soils

Soils used in this experiment were sampled from the vicinity of the area contaminated by the NFMW near Plovdiv, Bulgaria. Soils were collected from the surface (0- to 20-cm depth and 20- to 40-cm depth) of fields located at different distances (0.5 and 15.0 km) from the NFMW. The investigated soils are characterized by alkaline reaction, moderate $CaCO_3$ content, loamy texture, and moderate content of organic matter (Table 1). The soil samples were air dried, homogenized in an agitate mortar, and sieved. A fraction with particle size <1 mm was taken for analysis.

**TABLE 1**
**Soil Properties for Soil Sampled from the NFMW near Plovdiv**

| Classification | Distance from NFMW (km) | Depth (cm) | pH ($H_2O$) | Humus (%) | $CaCO_3$ (%) | Clay (%) |
|---|---|---|---|---|---|---|
| Calcaric | 0.5 | 0–20 | 7.72 | 2.19 | 7.30 | 29.35 |
| Fluvisol | | 20–40 | 7.75 | 1.82 | 7.50 | 32.23 |
| Calcaric | 15.0 | 0–20 | 7.47 | 1.54 | 8.70 | 12.71 |
| Fluvisol | | 20–40 | 7.62 | 1.01 | 8.94 | 13.82 |

## Plants

The winter rapeseed (*Brassica napus L. a napus*) was grown in the same regions situated at different distances (0.5 and 15.0 km) from the NFMW - Plovdiv. On reaching the "blossoming" stage, the rapeseed was gathered, and the content of Pb, Cd, Zn, and Cu in the different parts (stems, leaves, and inflorescences) used as fodder for rabbits was quantitatively determined. The samples were treated by the method of dry ashing.

## Rabbits

Rabbits of the breed Belgian Giant were bred in the experimental base of the "Stockbreeding" department at the Agricultural University - Plovdiv. They were fed twice a day (in the morning and in the evening) for a month, with fodder prepared specially for the purpose. Green fodder of rapeseed was added to the concentrated fodder (consisting mainly of barley). The rabbits were divided into two groups, each consisting of 20 animals. The first group was fed with rapeseed grown 0.5 km away from the NFMW (group A) and the second group was fed with rapeseed grown 15 km away from the NFMW (group B). Samples (bone, liver, kidney, muscle, and blood) from both groups of rabbits were collected when the animals were slaughtered. The samples were treated by the method of acid digestion.

## Heavy Metal Analysis

### *Sample Preparation*

#### Soils

1. *Total content* of heavy metals in soils was determined in accordance with the international standard for extraction of trace elements soluble in aqua regia ISO 11466[27]. Three grams of soil sample were decomposed on a sand bath heater for 3 h with 21 ml of concentrated hydrochloric acid (HCl) + 7 ml of concentrated nitric acid ($HNO_3$). After cooling the sample, the residue was transferred into a 50-ml flask and water was added up to the mark.
2. *The mobile heavy metal* contents have been determined in 0.005 *M* diethylentriaminepentaacetic acid (DTPA) and 0.1 *M* triethanolamine (TEA) buffered at pH 7.3[28]. Soil samples were shaken for 2 h at 20°C. After shaking, the soil-solution system was centrifuged and filtered. The ratio soil to liquid was 1:2 by weight to volume.
3. *Fractionation studies* — The distribution of heavy metals in the different forms and phases in which they occur in soil can be determined using sequential extraction procedures. Sequential extraction procedures provide information about the determination of the relative binding strength of the metal on various solid phases and about their potential reactivity under physicochemical environmental conditions. In the present study, a five-step Tessier sequential extraction scheme[29], separating exchangeable metals, metals bound to carbonates, metals bound to Fe-Mn oxides, metals bound to organic matter, and residual metals, was applied for the extraction of metals in soil samples for assessing the mobility of the metals.

### Plants

A 1-g sample was weighed into a quartz crucible and put into a furnace (400°C) until ashing occurred. After cooling to room temperature, 1 ml $HNO_3$ (1:1) was added, evaporated in a sand bath, and put again into the furnace (400°C). The procedures were repeated until the ash was white. It was finally dissolved in 2 ml 20% HCl (v/v), transferred into a graduated 25-ml flask, and brought to volume with doubly distilled water.

### Rabbits

1. *Liver, kidney, muscle, and blood* — A 1-g subsample (2 ml of blood) was digested with concentrated $HNO_3$ and hydrogen peroxide ($H_2O_2$). Digested samples were transferred into a graduated 10-ml flask and diluted with doubly distilled water.
2. *Bone* — A 5-g sample was placed into a quartz crucible and put into a furnace (400$^{o}$C) for 12 h. After cooling to room temperature, 10 ml $HNO_3$ was added and placed on a preheated hot plate and heated until its content was evaporated to 2–3 ml. The digest was cooled, filtrated, and diluted to 25 ml in a calibrated flask.

### *Equipment*

In order to determine the heavy metal content in the samples, an inductively coupled emission spectrometer (Jobin Yvon Horiba "ULTIMA 2", France) was used. A commercial multielement standard solution (Merck) with concentration 100 mg/l was used as a stock solution. The calibration standard solutions had the following concentrations: 0, 0.2, 0.5, 2.0, and 5.0 mg/l. The acidity of the standard and sample solutions was the same.

Certified reference materials (Contaminated Brickworks Soil - ERM CC135a; Apple Leaves - SRM1515; and Pig Kidney CRM 186 - BCR Reference Materials) were used for quality control. The results show acceptable agreement between the found and certified values for Cd, Cu, Pb; and Zn.

## RESULTS AND DISCUSSION

### Soils

The results (Table 2) showed that with increasing distance from the NFMW - Plovdiv and increasing the depth of the horizon, there was a very clear reduction of the heavy metal content in the soil.

In the soil samples taken from the region situated 0.5 km away from the NFMW, values for Pb exceeded the maximum permissible concentrations of 80 mg/kg for the country: 200.3 mg/kg in the 0- to 20-cm layer and 181.8 mg/kg in the 20- to 40-cm layer. The region that was 15 km away from the plants revealed that the concentration of Pb was reduced by more than 85%, and there was almost no difference between the two horizons. The results obtained for Zn and Cd were analogous. In the region of the NFMW,

**TABLE 2**
**Content of Pb, Cu, Zn, and Cd (mg/kg) in Soils Sampled from the NFMW**

| Distance from NFMW (km) | Depth (cm) | Pb | Cd | Zn | Cu |
|---|---|---|---|---|---|
| | | (x ± SD) | | | |
| 0.5 | 0–20 | 200.3 ± 6.0 | 12.2 ± 0.24 | 536.1 ± 4.7 | 95.7 ± 1.8 |
| | 20–40 | 181.8 ± 5.1 | 10.0 ± 0.18 | 434.0 ± 3.2 | 89.9 ± 1.7 |
| 15.0 | 0–20 | 24.6 ± 0.7 | 2.7 ± 0.04 | 33.9 ± 0.3 | 16.0 ± 0.3 |
| | 20–40 | 22.7 ± 0.7 | 2.5 ± 0.02 | 31.9 ± 0.3 | 13.9 ± 0.2 |
| **MPC** | | **80** | **2.5** | **340** | **260** |

*Note*: x, average value (mg/kg) from five repetitions; SD, mean standard deviation; MPC, maximum permissible concentration (approved for Bulgaria).

values of 536.1 mg/kg Zn and 12.2 mg/kg Cd were established, which considerably exceeded the maximum permissible concentrations; although in the more distant region, values of 33.9 mg/kg Zn and 2.7 mg/kg Cd were measured. Increasing the depth of the soil horizon resulted in a more intense decrease of content to 434.0 mg/kg Zn and 10.0 mg/kg Cd compared to that of the more distant region, while the differences between the two horizons were negligible. The quantity of Cu in the soil from the region of the NFMW was 95.7 mg/kg and at a distance of 15 km from the NFMW, it decreased considerably to 16.0 mg/kg. The contents of Cu in the soil from both regions of the investigation were considerably lower than the accepted Bulgarian maximum permissible concentration of 260 mg/kg. Increasing the depth of the horizon exhibited a weak trend of decreasing concentration of Cu in the soil.

Table 3 presents the results of the mobile forms of Pb, Cu, Zn, and Cd in the examined soils. The percentage contents of the mobile forms in proportion to the total quantity of the elements in the soils are also presented in Table 3. According to the results regarding the mobile forms of the metals, determined by DTPA, the mobile forms of Cd are 43.4% of its overall content, while for Pb, Cu, and Zn they are 34.8, 21.7, and 17.9%, respectively.

**TABLE 3**
**DTPA-Extractable Pb, Cu, Zn, and Cd (mg/kg) in Soils Sampled from NFMW**

| Distance from NFMW (km) | Depth (cm) | Pb | | Cd | | Zn | | Cu | |
|---|---|---|---|---|---|---|---|---|---|
| | | mg/kg (x ± SD) | %* | mg/kg (x ± SD) | % | mg/kg (x ± SD) | % | mg/kg (x ± SD) | % |
| 0.5 | 0–20 | 69.7 ± 1.8 | 34.8 | 5.3 ± 0.5 | 43.4 | 95.7 ± 2.2 | 17.9 | 20.7 ± 0.3 | 21.6 |
| | 20–40 | 48.9 ± 1.7 | 26.9 | 4.1 ± 0.2 | 41.1 | 68.6 ± 2.1 | 15.8 | 19.5 ± 0.2 | 21.7 |
| 15.0 | 0–20 | 4.9 ± 0.1 | 19.9 | 1.0 ± 0.02 | 37.0 | 3.7 ± 0.1 | 10.9 | 4.9 ± 0.2 | 30.6 |
| | 20–40 | 4.2 ± 0.1 | 18.5 | 0.9 ± 0.02 | 36.0 | 3.2 ± 0.1 | 10.0 | 4.3 ± 0.2 | 30.9 |

* DTPA-extractable/total content.

x, average value (mg/kg) from five repetitions; SD, mean standard deviation.

## *Fractionation of Soil*

Any metals derived from an anthropogenic source are strongly influenced by their form, phase, and oxidation state, and hence, bioavailability. Chemical soil tests are designed to extract a quantity of elements from the soil solids that correlate statistically to the size of the "available pool" in the soil defined by the quantity of elements taken by the plants[3]. Chemical extraction techniques provide a well-established means of identifying and characterizing different fractions of heavy metals in the soil[30].

Fig. 1 presents mean values (in %) of metal associated with different fractions (exchangeable metals, metals bound to carbonates, metals bound to Fe-Mn oxides, metals bound to organic matter, and residual metals).

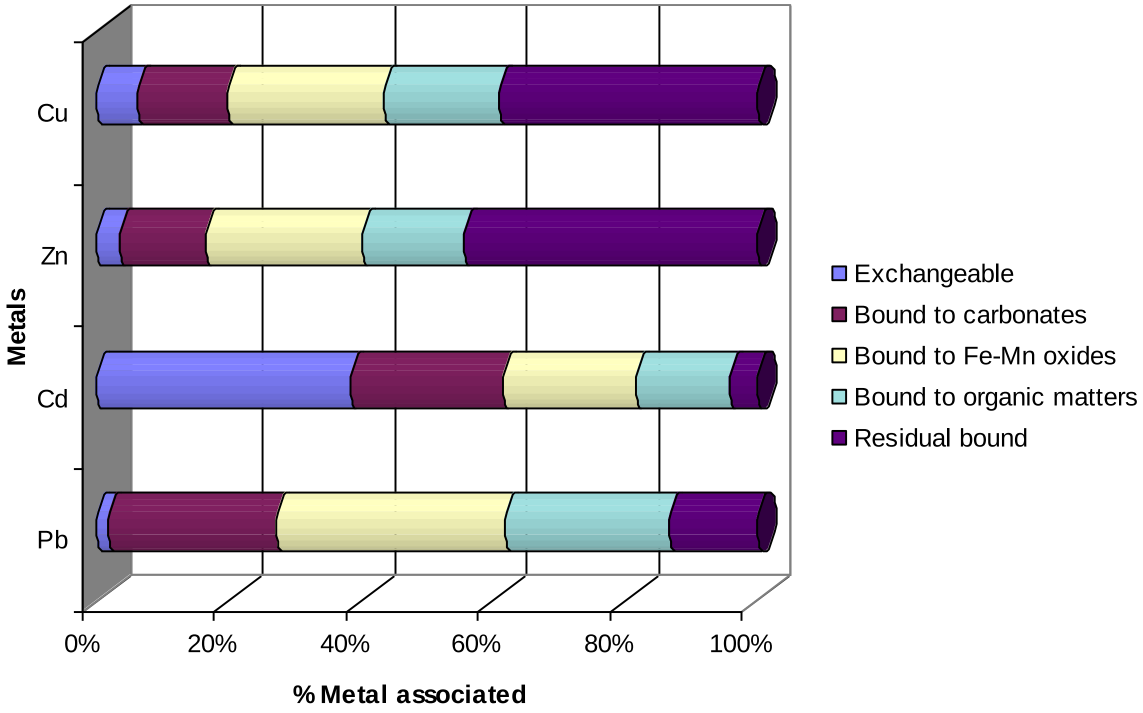

**FIGURE 1.** Association of metals with different fractions of soils of the study area.

Results of the geochemical partitioning using the Tessier scheme[29] revealed high concentrations of Cd to be associated with the mobile fraction of the soil. Most of the Pb, Cu, and Zn were bound with forms largely unavailable for plants, i.e., Fe-Mn oxides and residual fraction.

Because Pb binds strongly with organic matter and chemisorbs on oxides of Fe, Mn, and Al, it is a low-mobility metal in soil. Studies have reported that accumulation of Pb in plants occurs only with high concentration of Pb in soils[4,31]. As the soil Pb concentration was high, the vegetation is prone to accumulate Pb[1,3].

Based on the fractionation studies, the metals under study can be arranged in decreasing order of bioavailability: Cd > Pb > Cu > Zn.

These results are in accordance with those regarding the mobile forms of the metals as determined by means of DTPA.

## Rapeseed

Table 4 provides the results for the contents of heavy metals in the stems, leaves, and inflorescences of rapeseed used in fodder for the animals. All four elements were mainly concentrated in the inflorescences. In the inflorescences of the rapeseed grown 0.5 km away from the NFMW, Pb reached up to 10.70 mg/kg,

**TABLE 4**
**Content of Pb, Cd, Zn, and Cu (mg/kg) in Rapeseed in the "Blossoming" Stage**

| | Grown 0.5 km from NFMW (x ± SD) | Grown 15 km from NFMW (x ± SD) |
|---|---|---|
| | **Pb (MPC forage 5.0 mg/kg)** | |
| Stems | 1.73 ± 0.1 | 0.72 ± 0.02 |
| Leaves | 8.69 ± 0.5 | 1.42 ± 0.1 |
| Inflorescences | 10.70 ± 0.5 | 3.97 ± 0.2 |
| Concentrated fodder (barley) | 0.62 ± 0.02 | |
| | **Cd (MPC forage 0.5 mg/kg)** | |
| Stems | 0.11 ± 0.01 | 0.02 ± 0.01 |
| Leaves | 0.68 ± 0.02 | 0.16 ± 0.01 |
| Inflorescences | 1.40 ± 0.05 | 0.37 ± 0.02 |
| Concentrated fodder (barley) | 0.03 ± 0.01 | |
| | **Zn** | |
| Stems | 8.90 ± 0.3 | 3.55 ± 0.5 |
| Leaves | 27.80 ± 0.5 | 12.50 ± 0.4 |
| Inflorescences | 135.0 ± 1.1 | 72.97 ± 0.9 |
| Concentrated fodder (barley) | 24.91 ± 0.5 | |
| | **Cu** | |
| Stems | 4.94 ± 0.03 | 3.12 ± 0.04 |
| Leaves | 2.31 ± 0.04 | 1.66 ± 0.05 |
| Inflorescences | 7.40 ± 0.08 | 5.5 ± 0.03 |
| Concentrated fodder (barley) | 1.72 ± 0.04 | |

Note: x, average value (mg/kg) from five repetitions; SD, mean standard deviation; MPC, maximum permissible concentration (according to Directive 99/29/EC).

Cd to 1.40 mg/kg, Zn to 135.0 mg/kg, and Cu to 7.40 mg/kg, while the more distant region ascertained 3.97 mg/kg Pb, 0.37 mg/kg Cd, 72.97 mg/kg Zn, and 5.5 mg/kg Cu.

The content of heavy metals in the rapeseed leaves was lower compared to the inflorescences. In the leaves of rapeseed grown 0.5 km from the NFMW, Pb reached up to 8.69 mg/kg, Cd to 0.68 mg/kg, Zn to 27.80 mg/kg, and Cu to 2.31 mg/kg, while the more distant region ascertained 1.42 mg/kg Pb, 0.16 mg/kg Cd, 12.50 mg/kg Zn, and 1.66 mg/kg Cu.

Considerably lower values were established in the stems of rapeseed. In the stems of rapeseed grown 0.5 km from the NFMW, Pb reached up to 1.73 mg/kg, Cd to 0.11 mg/kg, Zn to 8.90 mg/kg, and Cu to 4.94 mg/kg., while the more distant region ascertained 0.72 mg/kg Pb, 0.02 mg/kg Cd, 3.55 mg/kg Zn, and 3.12 mg/kg Cu.

As the distance from the NFMW increased, there was a clear decrease in heavy metal contents in the stems, leaves, and inflorescences of the rapeseed.

The accumulation of Pb, Cd, and Zn in the rapeseed organs occurs in the following order: inflorescences > leaves > stems, for the polluted and nonpolluted soils. Only Cu is an exception to this, as it is the least found in the leaves.

The absorption of heavy metals by the rapeseed has an evident selective preference, as a strong affinity towards Zn is expressed, especially when considering the low percentage of its mobile forms. However, a direct connection between the quantities of the mobile forms and their content in plants is not observed.

## Rabbits

Table 5 summarizes the toxic metal levels in the liver, kidneys, muscles, bones, and blood of the rabbits that were fed with a fodder mixture containing rapeseed grown at 0.5 km (group A) and 15 km (group B) away from the NFMW.

**TABLE 5**
**Pb, Cd, Zn, and Cu Levels in Bone, Liver, Kidneys, Muscle (mg/kg wet weight), and Blood (mg/l) of Rabbits**

| Organs or Tissue | Fed with Rapeseed Grown 0.5 km from NFMW (Group A) (x ± SD) | Fed with Rapeseed Grown 15 km from NFMW (Group B) (x ± SD) | Pollution Factor (PF) |
|---|---|---|---|
| **Pb (MPC muscle - 0.1 mg/kg; liver and kidney - 0.5 mg/kg)** | | | |
| Bone | 1.0 ± 0.02 | 0.32 ± 0.01 | 3.12 |
| Liver | 0.65 ± 0.02 | 0.40 ± 0.01 | 1.63 |
| Kidneys | 3.89 ± 0.08 | 1.89 ± 0.05 | 2.80 |
| Muscle | 0.19 ± 0.01 | 0.11 ± 0.01 | 1.73 |
| Blood | 0.43 ± 0.01 | 0.36 ± 0.01 | 1.19 |
| **Cd (MPC muscle - 0.05 mg/kg; liver - 0.5 mg/kg; kidney - 1.0 mg/kg)** | | | |
| Bone | 0.025 ± 0.002 | 0.011 ± 0.001 | 2.27 |
| Liver | 0.023 ± 0.002 | 0.008 ± 0.001 | 2.88 |
| Kidneys | 0.077 ± 0.005 | 0.035 ± 0.002 | 2.20 |
| Muscle | nd | nd | |
| Blood | 0.013 ± 0.001 | nd | |
| **Zn (MPC muscle - 50.0 mg/kg; liver and kidney - 80 mg/kg)** | | | |
| Bone | 33.13 ± 0.5 | 54.50 ± 0.8 | 0.61 |
| Liver | 18.72 ± 0.01 | 26.65 ± 0.5 | 0.70 |
| Kidneys | 13.59 ± 0.3 | 1.32 ± 0.05 | 10.30 |
| Muscle | 9.99 ± 0.2 | 2.19 ± 0.1 | 4.65 |
| Blood | 3.14 ± 0.1 | 3.76 ± 0.1 | 0.86 |
| **Cu (MPC muscle - 5.0 mg/kg; liver and kidney - 60 mg/kg)** | | | |
| Bone | 0.65 ± 0.01 | 0.71 ± 0.01 | 0.91 |
| Liver | 1.48 ± 0.03 | 1.47 ± 0.04 | 1.01 |
| Kidneys | 1.44 ± 0.03 | 1.70 ± 0.04 | 0.85 |
| Muscle | 0.66 ± 0.01 | 0.11 ± 0.01 | 6.00 |
| Blood | 0.45 ± 0.01 | 0.52 ± 0.01 | 0.87 |

*Note*: x, average value (mg/kg) from five repetitions; SD, mean standard deviation; MPC, maximum permissible concentration (approved for Bulgaria).

In both groups of animals, Cd levels were significantly higher in the kidneys than in the liver and bone. Levels in kidneys and liver were significantly higher than in blood and muscle, as Cd in muscle was below the detection limit. In the blood and all tissues analyzed (Table 4), rabbits from group A displayed significantly higher Cd levels than the rabbits from group B. Pollution factors (PF), calculated as ratios of the metal levels in the rabbits from group A to the metal levels in rabbits from group B, were 2.88, 2.27, and 2.20 for liver, bone, and kidneys, respectively.

Several studies have shown that Cd concentrates more in the kidneys than in the liver[20,26,32,33,34,35,36,37,38,39].

The higher concentration of Cd in the kidney tissue is due to the detoxification function of the organ where these metals are accumulated[40,41]. Animals exposed to Cd accumulate it in their liver and kidneys, as their free protein-thiol group content leads to a strong fixation of heavy metals. Despite the excretory mechanism for such metals, which is based on low molecular compounds with -SH groups, vertebrates could not develop these mechanisms during the period of evolution to the extent necessary for today's anthropogenic sources of pollution[35]. The herbivores of terrestrial fauna, birds as well as mammals, demonstrate generally higher renal Cd than carnivores, since vegetation is contaminated by aerial deposition or by absorption of Cd from the soil[42].

A similar trend was also observed with Pb levels. The most marked effect of pollution on Pb levels was seen in the bone (PF = 3.12) and kidneys (PF = 2.80), and to lesser extent in the muscle (PF = 1.73) and liver (PF = 1.63). The difference between Pb accumulation in the blood of rabbits from group A and group B is negligible.

As can be seen from Figs. 2 and 3, the trend of Pb and Cd distribution after the introduction of polluted fodder remains unchanged, as the increase of their contents is proportional to the initial levels. The contents of these elements in the organs of the rabbits from group A and group B exhibit a linear relationship, and $R^2$ is more than 0.95. It is clearly evident that the quantity of accumulation of both elements in all animals' organs is directly associated with diet composition.

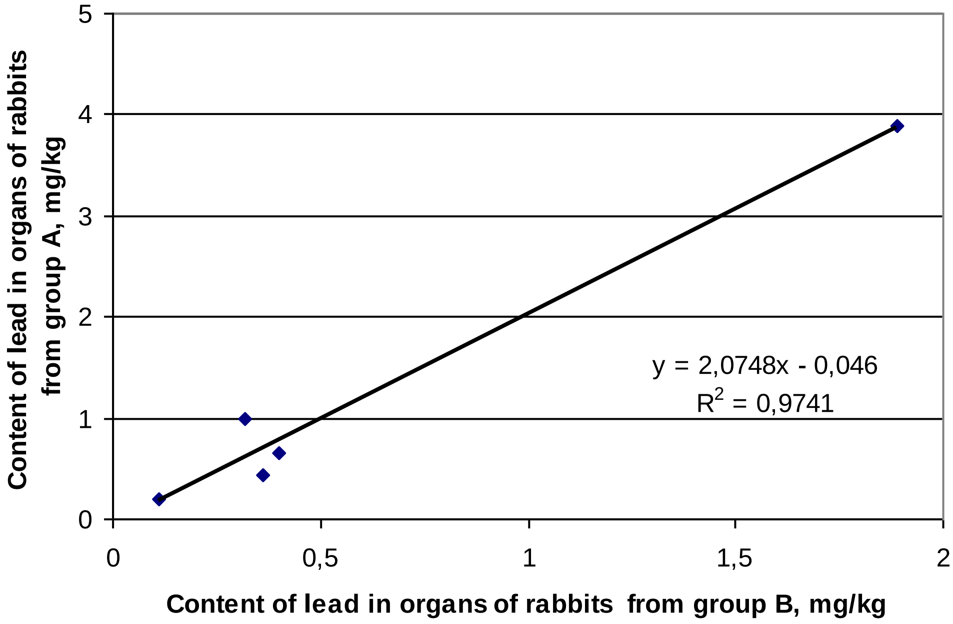

**FIGURE 2.** Relationship between the content of Pb in the organs of the rabbits from group A and group B.

However, this result is the converse of Cu and Zn, where the content of Zn in the bones, liver, and blood is comparatively high, and is independent of diet composition. Further, contrary to expectations, its content in the bones, liver, and blood of the rabbits from group A is less than the content in the rabbits from group B. However, a different result is observed in the kidneys and muscle, where PF is 10.3 and 4.65, respectively. Evidently, in addition to the content of heavy metals in the food of the rabbits, there is a further factor that affects the absorption and distribution of heavy metals within the organs of the animals.

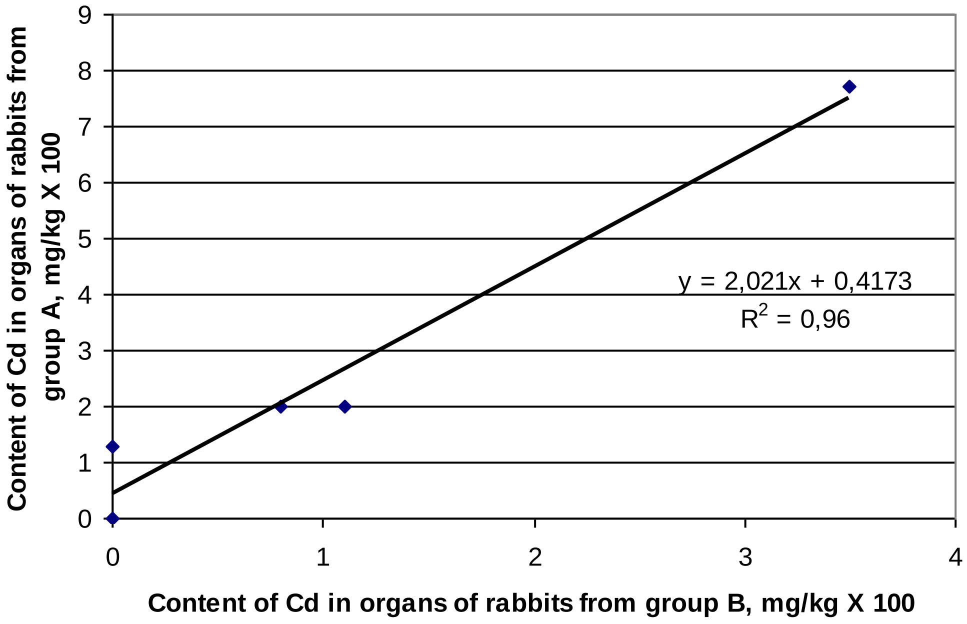

**FIGURE 3.** Relationship between the content of Cd in the organs of the rabbits from group A and group B.

Although the accumulation of Cu in the organs of the rabbits, in terms of absolute values, is considerably less, a similar trend nevertheless exists, as the diet composition likewise has no effect on the concentration of Cu in the bones, liver, and blood. PF in the muscle is 6.0. In contrast to Zn, however, in this case there is no observed accumulation of the element in the kidneys.

The results of this study clearly indicate that environmental contamination has a significant impact on heavy metal concentrations in rabbits. Similar results have been reported previously for livestock (cattle, beef, sheep, goat) in other polluted environments, including areas in the vicinity of Zn refineries[43] and metalliferous areas[24,25,44,45,46,47].

Only broad comparisons can be made between the results of the present study and data reported previously, as there are essentially no existing data on heavy metals levels in rabbit tissue.

The effects of pollution on toxic metal levels in rabbits can be compared with data reported elsewhere for the other animals on the basis of pollution factors (PFs). PF values have been widely used in monitoring studies[48,49] for estimation of the proportion of tissue metal content that has anthropogenic origin. Taking PF values into account, the contribution of pollution to Cd levels in our experiment for rabbits was equivalent to that of other studies of livestock reared close to pollution sources[31,43,46]. This effectively confirms suggestions that the kidney is the primary Cd storage organ in animals subject to chronic low-level Cd exposure[50]. For Pb, the effect of pollution in rabbits was more pronounced than in previous studies[31,43,46].

It was found that there was a well-expressed impact of the level of Cd contamination on the absorption of essential trace metals (Zn and Cu) and their accumulation into some of the organs of the animals. Rabbits nourished with rapeseed grown 0.5 km from the NFMW showed significantly lower hepatic Zn concentrations than rabbits from group B. Similar results have been reported for livestock raised close to Zn refineries[43,44] as well as in Zn-polluted areas[24,49]. In fact, the low Zn concentrations in livestock from the industrialized area could also be attributed to the higher Cd exposure. Cd causes reductions in both intestinal Zn absorption and hepatic Zn reserves in cattle[51], as a result of competition for the cation-binding sites of metallothionein[7].

We observed apparently similar effects on the other essential metal, Cu. Cu levels in the liver of rabbits from groups A and B were almost identical, whereas in blood, kidneys, and bone, they were significantly lower in the animals from group A. Decreases in hepatic Cu levels have been found in livestock from metalliferous and mining areas[43,46,49], and are assumed to be related to chemical interactions between Cd and Cu. This chemical interaction appears to be very relevant, since hepatic Cu levels sometimes drop into the deficiency range in areas of severe Cd pollution[43,44,49,52].

## CONCLUSIONS

A comparative research study on the bioavailability of Pb, Cd, Zn, and Cu in the soil-plant-animal system was undertaken. This involved an investigation of the connection between the total quantity and the mobile forms of Pb, Cd, Zn, and Cu in soils of varying levels of contamination; the transition of these metals into rapeseed; and their ingestion by rabbits fed with a rapeseed-dominant food mixture.

It was established that:

1. The uptake of heavy metals by rapeseed has an evident selective preference, as a strong affinity towards Zn is expressed. The accumulation of Pb, Cd, Zn, and Cu in the organs of the rapeseed occurs in the following order: inflorescences > leaves > stems. A direct relationship between the quantity of the mobile forms and their accumulation in the plants was not found.
2. Environmental contamination has a significant impact on heavy metal concentrations and distribution, as all four trace elements are primarily accumulated in the kidneys and liver. The characteristics of Pb and Cd distribution after the introduction of polluted fodder remain unchanged, as the increase of their content is proportional to the initial value. However, this is not valid for Cu and Zn.
3. There is a well-expressed impact of the level of Cd contamination on the absorption of essential trace metals (Zn and Cu) and their accumulation into some of the internal organs of the animals.
